# From density to spatial function: spatial multi-omics, composite biomarker design, and therapeutic engineering of tertiary lymphoid structures in solid tumors

**DOI:** 10.3389/fimmu.2026.1865949

**Published:** 2026-10-05

**Authors:** Xuchen Wang, Lin Lin

**Affiliations:** 1 The Sixth Clinical Medical College of Guangzhou Medical University (Qingyuan People’s Hospital), Qingyuan, China; 2 Department of Oncology, The First Affiliated Hospital of Shenzhen University, The Second People’s Hospital of Shenzhen, Shenzhen, China

**Keywords:** B cells, composite biomarkers, high endothelial venules, immunotherapy, multiplexed imaging, spatial transcriptomics, tertiary lymphoid structures, tumor microenvironment

## Abstract

**Background:**

Tertiary lymphoid structures (TLSs) have emerged as clinically relevant immune niches associated with improved survival and enhanced response to immune checkpoint inhibitors across multiple solid tumors. However, translational evaluation remains dominated by density-based scoring, which inadequately captures the functional heterogeneity of histologically similar lymphoid aggregates. This review synthesizes recent advances in spatial biology that support a shift from counting TLSs to interpreting them as spatially organized immune microenvironmental units embedded within local tumor ecosystems.

**Main body:**

Evidence from spatial transcriptomics, multiplexed imaging, single-cell profiling, computational pathology, and spatial-proteomic approaches converges on four coupled requirements for productive TLS function: stromal licensing through lymphotoxin-dependent chemokine circuits, vascular competence mediated by specialized high endothelial venules, helper T-cell programs that bridge follicular and cytotoxic immunity, and a permissive metabolic context that enables maturation. Spatial studies further show that regulatory infiltration, suppressive plasma-cell programs, and metabolic disruption can decouple structural presence from effective antitumor immunity. Across tumor types, these insights indicate that biomarker performance improves when TLS density is integrated with maturity, anatomical location, HEV coupling, immune composition, and treatment context rather than used as a standalone metric. We further discuss how spatial analyses reveal actionable pathways for inducing or reprogramming TLSs and outline a staged, module-matched composite biomarker workflow combining digital pathology, targeted multiplexed imaging, and focused molecular or metabolic augmentation.

**Conclusions:**

TLSs should be regarded as spatially organized, functionally heterogeneous immune microenvironmental units rather than biologically equivalent lymphoid aggregates. A spatial-functional framework helps explain productive, arrested, and suppressive TLS states, provides a basis for next-generation composite biomarkers, and supports module-matched therapeutic strategies tailored to the stromal, vascular, helper-cell, or metabolic bottleneck limiting TLS function in individual tumors. The immediate next step is prospective validation of a minimal TLS panel across defined treatment contexts.

## Background

Tertiary lymphoid structures (TLSs) are ectopic lymphoid aggregates that arise in chronically inflamed non-lymphoid tissues and were historically characterized through hematoxylin and eosin (H&E) morphology alone. Over the past fifteen years, they have been reinterpreted as biologically meaningful immune niches whose organization shapes tumor progression and response to immunotherapy. A pivotal series of studies first established this relevance: Dieu-Nosjean and colleagues demonstrated that intratumoral lymphoid structures predict long-term survival in non-small-cell lung cancer (NSCLC) (1); Coppola and colleagues identified analogous structures in colorectal carcinoma through immune gene-array profiling (2); and landmark 2020 *Nature* reports linked B-cell–rich TLSs to durable responses to immune checkpoint inhibitor (ICI) therapy in melanoma and sarcoma cohorts (3–6). The subsequent proliferation of immune-oncology trials intensified a central clinical question: which TLSs are biologically productive, and which are merely histologically visible (7–10)?

This distinction matters because TLSs are not monolithic. Loose lymphocytic aggregates, follicle-like clusters with follicular dendritic cell (FDC) networks, and germinal-center–bearing structures are often lumped together under a single diagnostic term despite representing functionally distinct biology. Conventional histology quantifies density and coarse anatomical position but cannot resolve chemokine gradients, endothelial specialization, clonal architecture, or the metabolic barriers that separate productive from arrested maturation. Canonical TLSs are organized around a central B-cell follicle, often with a germinal center, and an adjacent zone of CD4^+^ helper T cells. Dendritic cells, fibroblastic reticular-cell networks, extracellular matrix, and high endothelial venules (HEVs) provide antigen-presenting, structural, and vascular support, while CD8^+^ T cells and natural killer cells supply local effector activity (**Figure 1**). Density counts collapse all of these features into a single metric; spatial readouts preserve them. Recent comprehensive reviews acknowledge this heterogeneity (7–11), yet they tend to synthesize TLS biology broadly rather than dissecting how functionally distinct niches actually assemble, stall, or deviate in human solid tumors.

**Figure 1:**
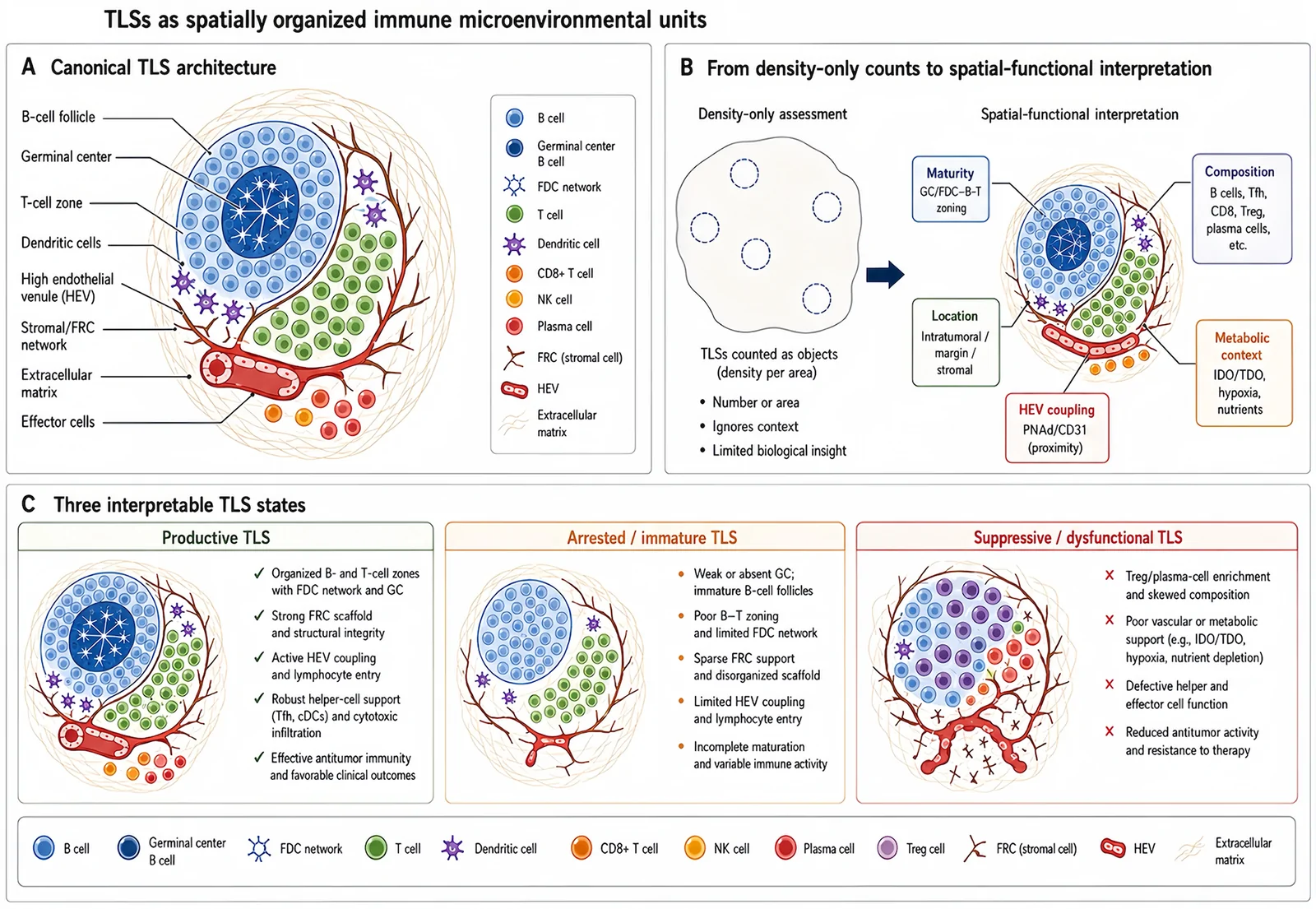
TLSs as spatially organized immune microenvironmental units. Panel **(A)** illustrates canonical TLS architecture as a compartmentalized immune niche containing a B-cell follicle with germinal center, adjacent T-cell zone, dendritic cells, high endothelial venule (HEV), stromal/fibroblastic reticular cell (FRC) network, extracellular matrix, and effector-cell compartment. Panel **(B)** contrasts density-only assessment with spatial-functional interpretation by integrating maturity, anatomical location, HEV coupling, immune composition, and metabolic context. Panel **(C)** compares productive, arrested/immature, and suppressive/dysfunctional TLS states according to organization, vascular competence, immune composition, metabolic support, and expected antitumor function. Cell, vascular, stromal, and matrix symbols are identified in the figure keys. The arrow in panel **(B)** indicates the shift from density-only assessment to spatial-functional interpretation. In panel **(C)**, green check marks label productive features, orange bullets label arrested or immature features, and red crosses label suppressive or dysfunctional features. A plus sign (+) denotes marker-positive cells. cDC, conventional dendritic cell; CD8, cluster of differentiation 8; CD31, cluster of differentiation 31; FDC, follicular dendritic cell; FRC, fibroblastic reticular cell; GC, germinal center; HEV, high endothelial venule; IDO, indoleamine 2,3-dioxygenase; NK, natural killer; PNAd, peripheral node addressin; TDO, tryptophan 2,3-dioxygenase; Tfh, T follicular helper cell; TLS, tertiary lymphoid structure; Treg, regulatory T cell.

A targeted, non-systematic literature search was conducted between January 2016 and March 2026 using PubMed, Web of Science, and publisher repositories. Search terms combined “tertiary lymphoid structure(s)” and “tumor-associated tertiary lymphoid structure(s)” with “spatial transcriptomics,” “single-cell,” “single-nucleus,” “multiplex imaging,” “multiplex immunofluorescence,” “imaging mass cytometry,” “digital spatial profiling,” “digital pathology,” “high endothelial venule,” “CXCL13,” “immunotherapy,” and cancer-type qualifiers. Peer-reviewed solid-tumor studies with spatially detailed data or direct translational implications were prioritized; landmark mechanistic and immune-checkpoint-blockade studies were included regardless of publication date. Studies primarily focused on non-solid tumors, autoimmune or infection-associated TLSs, transplantation, conference abstracts without full peer-reviewed publication, preprints, and non-English publications were not used as primary evidence. When multiple reports appeared to use overlapping cohorts, the most methodologically detailed or most recent report was prioritized, and overlapping evidence was discussed qualitatively rather than treated as independent validation. This is a narrative review and does not constitute a systematic review or meta-analysis; effect-size estimates cited are drawn from published meta-analyses and are used for framing rather than pooled re-analysis.

The present review is focused and translational in scope. Five questions structure the argument: Why does TLS biology require spatial interpretation? What can current spatial multi-omics platforms genuinely resolve, and where do they mislead? Which cellular circuits drive or arrest maturation? What patterns emerge across recent tumor-specific studies, including historically immunotherapy-refractory cancers? And how can spatial insight be translated into composite biomarkers and module-matched therapeutic engineering?

## Why TLS biology requires a spatial frame of analysis

A density-only TLS score obscures the first clinically relevant question: where is the structure situated relative to tumor nests, invasive margins, vascular beds, fibroblastic tracks, and zones of hypoxia or necrosis? Intratumoral TLSs are consistently associated with stronger lymphocyte trafficking and more direct contact with malignant cells, whereas peritumoral aggregates may reflect immune organization that remains spatially decoupled from tumor elimination. Classic NSCLC cohorts first suggested this distinction (1), and more recent spatial characterizations of lung, gastrointestinal, and hepatobiliary malignancies have made it impossible to ignore (12–16).

Maturity adds a second interpretive layer. Germinal-center (GC) formation, FDC networks, plasma-cell differentiation, and HEV development collectively indicate that a TLS is functioning as an adaptive immune niche rather than existing as a transient inflammatory cluster. Even maturity markers, however, require context. Structures infiltrated by regulatory T cells (Tregs) or containing immunosuppressive plasma-cell programs behave fundamentally differently from B-cell–rich follicles coupled to HEVs and supported by stem-like or T-follicular-helper–like T cells. Spatial studies in lung, cervical, and nasopharyngeal cancers have repeatedly illustrated that identical histological labels can mask opposite functional programs (12, 17–19).

Spatial analysis also explains why cell-type composition cannot be reliably inferred from bulk transcriptomic data. A high CXCL13 signal, for example, can originate from T cells, cancer-associated fibroblasts (CAFs), or mixtures of both; plasma-cell gene signatures can reflect local antitumor differentiation in one context and immunosuppressive antibody production in another (12, 17). Without spatial resolution, such signals become cohort averages that obscure mechanism. Accordingly, modern TLS interpretation depends increasingly on neighborhood-level analysis rather than on bulk deconvolution (7, 9, 20).

This TLS-centered view is consistent with, but narrower than, the broader spatial tumor-microenvironment literature. Cellular-neighborhood analyses have shown that antitumor immunity depends not only on which cell states are present but also on how they are assembled into recurrent local communities. For example, CODEX profiling of 140 tissue regions from 35 advanced colorectal cancers using 56 protein markers identified conserved cellular neighborhoods whose coordination at the invasive front was associated with antitumor immune organization and outcome (21).

TLSs can therefore be viewed as a specialized form of spatial immune neighborhood within the tumor ecosystem. Unlike many immune-rich neighborhoods, however, TLSs have recognizable B- and T-cell zoning, stromal scaffolds, vascular access through HEVs, and metabolic constraints that determine whether the structure behaves as an adaptive immune niche. The contribution of the present review is to translate that broader cellular-neighborhood concept into an operational TLS framework that can be measured, scored, and eventually linked to therapeutic decisions.

Framed differently, spatial interpretation does more than add detail and it prevents categorical misclassification. An identical TLS count may represent HEV-coupled, GC-bearing intratumoral follicles in one specimen and Treg-enriched, peritumoral, metabolically constrained aggregates in another. These are not equivalent biological states, and treating them as such in biomarker models has contributed to the heterogeneous effect sizes observed across TLS literature (9, 10, 22, 23). The framework summarized in **Figure 1C** formalizes this reframing: density, composition, location, vasculature, and metabolism jointly determine whether an aggregate functions as a productive immune neighborhood or persists as a bystander lymphoid cluster.

## What spatial multi-omics adds and where it can mislead

Current spatial platforms interrogate TLS biology at complementary rather than interchangeable resolutions. For TLS research, the operative question is not which technology is “best” in the abstract but which source of error each platform corrects. Histology is strong for structural presence, coarse topography, and some maturity features; spatial transcriptomics resolves chemokine, stromal, and metabolic fields; multiplexed imaging establishes anatomical zoning and HEV coupling; single-cell transcriptomics sharpens cell-state annotation; and computational pathology scales these measurements to clinically meaningful cohorts. Cross-study disagreement frequently reflects a mismatch between the question posed and the platform used. An integrated workflow spanning sample acquisition and clinical annotation, complementary spatial profiling, computational integration, biological and clinical interpretation, and orthogonal validation with clinical translation provides a template against which individual study designs can be benchmarked (**Figure 2**).

**Figure 2:**
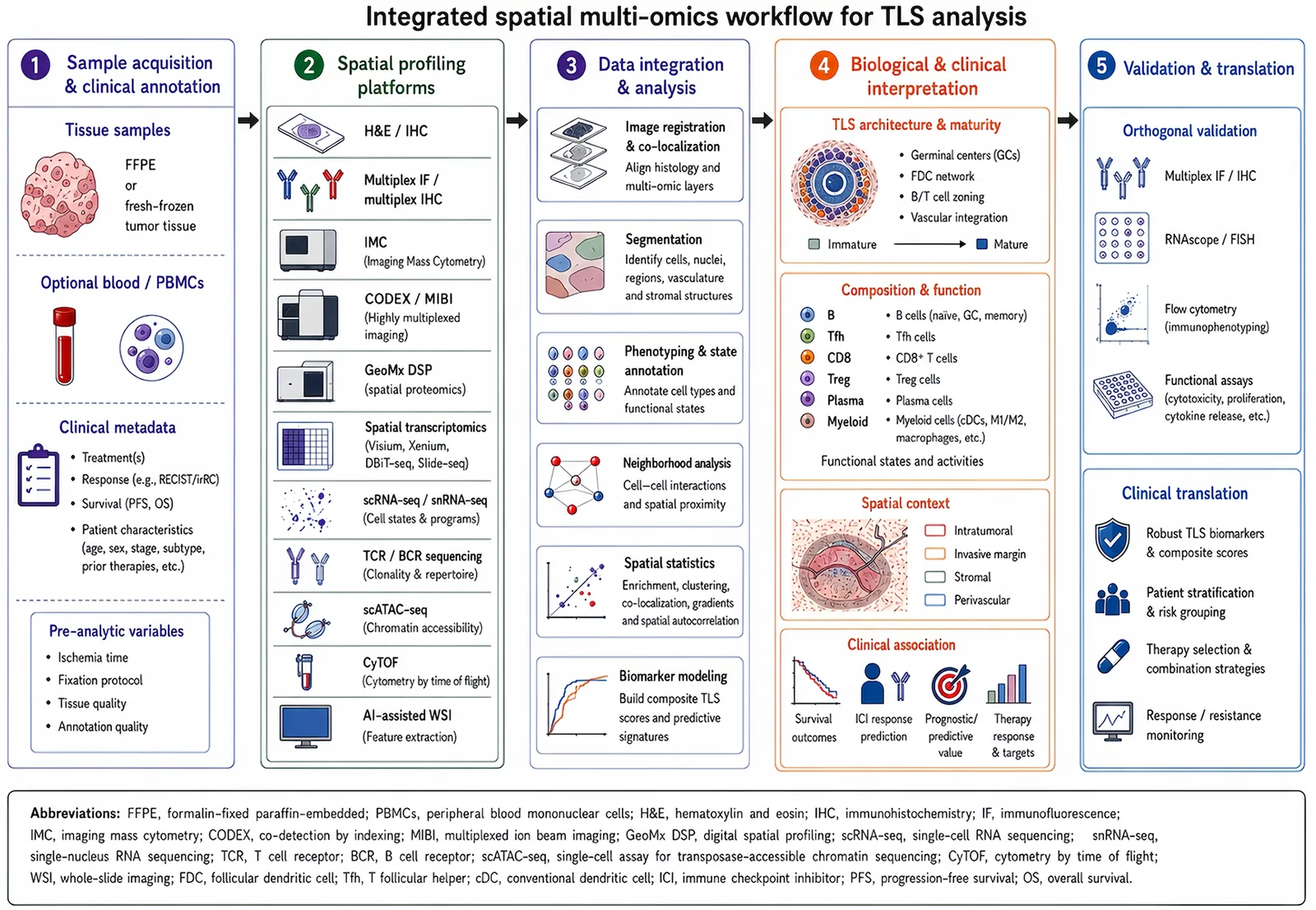
Integrated spatial multi-omics workflow for TLS analysis. The five-stage workflow begins with sample acquisition and clinical annotation, proceeds through named profiling platforms (H&E/IHC, multiplex IF/IHC, IMC, CODEX/MIBI, GeoMx DSP, spatial transcriptomics including Visium, Xenium, DBiT-seq, and Slide-seq, scRNA-seq/snRNA-seq, TCR/BCR sequencing, scATAC-seq, CyTOF, and AI-assisted whole-slide image analysis), and then integrates image registration, segmentation, phenotyping/state annotation, neighborhood analysis, spatial statistics, and biomarker modeling. Biological interpretation includes TLS architecture and maturity, composition and function, spatial context, and clinical association, followed by orthogonal validation and clinical translation. FFPE, formalin-fixed paraffin-embedded; PBMCs, peripheral blood mononuclear cells; IF, immunofluorescence; IHC, immunohistochemistry; IMC, imaging mass cytometry; DSP, digital spatial profiling; FISH, fluorescence *in situ* hybridization; WSI, whole-slide imaging. The numbered circles and connecting arrows denote the five sequential workflow stages. In the architecture panel, the light-to-dark scale denotes progression from immature to mature TLS organization; the outline colors in the spatial-context panel correspond to the four labeled anatomical locations. All assay, analysis, and clinical-translation icons are paired with their labels in the figure. A plus sign (+) denotes marker-positive cells. AI, artificial intelligence; B/T, B-cell/T-cell; BCR, B-cell receptor; cDC, conventional dendritic cell; CD8, cluster of differentiation 8; CODEX, co-detection by indexing; CyTOF, cytometry by time of flight; DBiT-seq, deterministic barcoding in tissue for spatial omics sequencing; FDC, follicular dendritic cell; GC, germinal center; H&E, hematoxylin and eosin; ICI, immune checkpoint inhibitor; irRC, immune-related response criteria; M1/M2, macrophage polarization states; MIBI, multiplexed ion beam imaging; OS, overall survival; PFS, progression-free survival; RECIST, Response Evaluation Criteria in Solid Tumors; scATAC-seq, single-cell assay for transposase-accessible chromatin sequencing; scRNA-seq, single-cell RNA sequencing; snRNA-seq, single-nucleus RNA sequencing; TCR, T-cell receptor; Tfh, T follicular helper cell; TLS, tertiary lymphoid structure; Treg, regulatory T cell.

### Spatial transcriptomics and the problem of mixed neighborhoods

Spot-based spatial transcriptomics introduced a workable compromise between transcriptomic breadth and preserved tissue context. The original Spatial Transcriptomics platform and its successors, including Slide-seq, Slide-seqV2, Visium, DBiT-seq, and HDST, provide broad transcriptomic maps of tissue sections, enabling systematic detection of chemokine gradients, stromal programs, and immune-rich neighborhoods that conventional immunohistochemistry cannot quantify at scale (24–29). By contrast, targeted *in situ* platforms such as Xenium, RNAscope, and FISH provide higher-resolution validation of selected gene programs rather than unbiased genome-wide profiling. For TLS research, this was a decisive conceptual shift: stromal, endothelial, B-cell, and T-cell features could now be analyzed within a single tissue plane rather than inferred across serial sections (**Figure 2**, stage 2).

The limitation is equally clear. Spot-level methods average signal across multiple cells, a problem that becomes especially acute in compact follicles where HEVs, fibroblasts, dendritic cells, B cells, and plasma cells occupy overlapping capture radii. Near-cellular platforms and statistical deconvolution partially mitigate this issue but do not eliminate it. Consequently, spot-based TLS studies are strongest for questions concerning regional programs, including maturation gradients, tumor-adjacent metabolic fields, and chemokine ecosystems, rather than precise cell–cell contact maps (27–29).

Recent high-density platforms extend spatial transcriptomic resolution and tissue coverage. Stereo-seq (spatial enhanced resolution omics-sequencing) uses DNA nanoball-patterned arrays with approximately 220-nm capture spots and center-to-center spacing as fine as 500 nm to profile large tissue areas (30). Visium HD uses continuous 2-µm barcoded squares and has mapped immune-cell niches in formalin-fixed paraffin-embedded colorectal cancer tissue, predominantly using 8-µm bins and reference-based deconvolution (31). Capture-feature dimensions are therefore distinct from effective cellular resolution: transcript recovery, binning, and segmentation determine whether neighboring cells can be resolved.

Recent human cohorts illustrate this strength. In hepatocellular carcinoma (HCC), Stereo-seq-based near-single-cell spatial profiling by Tang and colleagues resolved immature TLS trajectories and linked an unfavorable trajectory to tumor-derived tryptophan metabolism, demonstrating that inhibition of this pathway can synergize with anti–PD-1 therapy in murine models (12). In gastric cancer and nasopharyngeal carcinoma, integrated single-cell and spatial transcriptomic work defined TLS-associated immune states, spatial localization patterns, and proximity to malignant aggregates (11, 32). Pan-cancer spatial transcriptomic integration has extended the same logic, suggesting that TLS maturation follows recurring spatial rules despite tissue-specific variation (33). The analytical pipeline shared by these studies—image registration, cell segmentation, compositional profiling, neighborhood analysis, and spatial statistical modeling—is schematized in **Figure 2**, stage 3.

### Multiplexed imaging and spatial proteomics

Where transcriptomics expands the molecular field, multiplexed imaging and spatial proteomics resolve anatomy. Multiplexed ion beam imaging, co-detection by indexing, imaging mass cytometry, multiplex immunofluorescence, and GeoMx digital spatial profiling are particularly informative for TLS questions that hinge on vascular or subcellular precision: whether PNAd-positive HEVs are embedded in or adjacent to the follicle, whether B- and T-cell zones are properly segregated, whether Tregs or myeloid suppressor states occupy the TLS core or rim, and whether plasma cells infiltrate tumor nests or remain within lymphoid compartments (29, 34–36).

The practical advantage of these platforms is not merely higher plexity but direct architectural adjudication. Immunohistochemistry- or multiplex-immunofluorescence-based mature-TLS scoring has already been used to test whether mature TLSs predict ICI benefit independently of PD-L1 expression (37), HEV-focused IHC studies in colorectal cancer and neoadjuvant NSCLC have linked HEV abundance or proportion to prognosis and treatment response (16, 38), and high-dimensional spatial studies in ovarian and other tumors show that TLS-related activity is conditioned by anatomical site, stromal context, and surrounding immune-cell neighborhoods (21, 39, 40). These examples illustrate why revised evidence maps should name actual platforms and assay readouts rather than using generic labels such as “spatial profiling” or “multi-omics profiling”.

Spatial-proteomic platforms also expose a key limitation of transcript-only TLS interpretation. A CXCL13- or CCL19/CCL21-rich region may suggest lymphoid organization, but only protein-level tissue imaging can establish whether a follicle has a coherent FDC network, whether CD3/CD4/CD8 cells occupy appropriate zones, whether PNAd/CD31-positive vessels are spatially coupled to the follicle, and whether suppressive Treg, macrophage, or plasma-cell states are positioned in ways likely to alter local function. Conversely, a high-plex imaging panel is only as informative as its design; panels that omit FDC markers, HEV markers, T-cell helper markers, or metabolic/suppressive markers may classify TLS architecture accurately but miss the functional state that determines clinical meaning.

For this reason, spatial-proteomic data are most useful when integrated with H&E review, single-cell annotation, and targeted RNA or protein readouts. In the revised evidence map (**Table 1**), platform type, sample or cohort information, TLS-definition markers, endpoints, and reported limitations are separated so that readers can distinguish structural pathology, high-dimensional protein mapping, spatial transcriptomics, and computational pathology rather than treating them as interchangeable evidence streams.

**Table 1:** Evidence map of representative spatial or spatially integrated TLS studies in solid tumors.

Tumor/context	Study/refs	Sample/cohort context	Named platform(s)	TLS definition/readouts	Endpoint/key finding	Main limitation/caution
NSCLC	Dieu-Nosjean et al.; Goc/Germain et al. (1, 13, 14)	Resected NSCLC cohorts	Histopathology and IHC/immune-contexture analysis	Intratumoral lymphoid structures; DC-LAMP+ mature dendritic-cell-rich TLS; B-cell TLS contexture	TLS-rich NSCLC associated with long-term survival and protective antitumor immunity	Pre-spatial multi-omics evidence; TLS cutoffs, maturity criteria, and location definitions vary across cohorts
NSCLC	Elfving et al.; Zhang et al.; Matsumoto et al. (15, 38, 41)	Resected NSCLC; neoadjuvant immunochemotherapy cohort; durable ICI-response cohort	H&E/IHC, digital pathology or spatial-distribution analysis, HEV-focused IHC, single-cell spatial analysis	TLS location, HEV abundance, and ID1-high endothelial cells in TLSs	TLS topography, HEV-rich TLSs, and candidate endothelial states refine prognosis or ICI-response interpretation	Exact thresholds for “intratumoral”, HEV positivity, and endothelial substates require prospective harmonization
NSCLC	Du et al. (63)	ORIENT-11, OAK, POPLAR, and TCGA-LUAD; 171 ORIENT-11 patients had PD-L1 TPS and RNA-seq data	Bulk RNA-seq; ssGSEA scoring of 17 TLS gene signatures; PD-L1 TPS by 22C3 assay	TLS signature 3; transcriptomic TLS-high/TLS-low groups	TLS signature 3 predicted first-line chemoimmunotherapy outcomes and complemented PD-L1 testing	Transcriptomic signature; does not directly measure TLS spatial maturity or zoning; PD-L1-negative subgroup was small
Melanoma	Cabrita et al.; Lynch et al. (5, 64)	Checkpoint-blockade and metastatic melanoma cohorts	Pathology, transcriptomic/immune profiling, and B-cell heterogeneity analysis	Organized B-cell-rich TLS niches and TLS-associated B-cell states	TLS/B-cell-rich niches associate with ICI response and survival	B-cell abundance, TLS maturity, and TLS function should not be treated as interchangeable
Hepatocellular carcinoma	Tang et al.; Li et al.; Jiang meta-analysis (12, 23, 65)	HCC specimens and ICI-related solid-tumor evidence; exact HCC sample size not reported in this table	Histology/IHC, spatial transcriptomics, metabolic-pathway analysis, and meta-analytic synthesis	Conforming vs deviating TLS trajectories; HEV abundance; IDO/TDO-tryptophan/kynurenine programs	Conforming TLSs associate with antitumor immunity and anti-PD-1 responsiveness; high TLS density predicts ICI response across solid tumors	Metabolic readouts require compact clinical assays; meta-analysis captures density more than spatial-functional state
Gastric cancer	Wang et al.; Wang et al. (32, 58)	30 GC specimens stratified by TLS spatial localization; 25 profiled by scRNA-seq and five by ST-seq; anti-PD-1 GC outcome cohort	10x scRNA-seq, TCR-seq, 10x Visium ST-seq, H&E, immunofluorescence, and multiplex immunostaining	iTLS/pTLS/dTLS patterns; CXCL13+ CD4/CD8 T-cell-like clusters; germinal-center B cells; ACKR1+ HEV-related interactions	TLS spatial pattern and CXCL13-linked T-cell programs improve prognosis and immunotherapy-response stratification	External validation and assay-specific cutoffs are still needed before clinical deployment
Colorectal/rectal cancer	Coppola et al.; Zhan et al.; Schürch et al.; Wang et al. (2, 16, 21, 66)	Primary CRC; LARC cohort with 242 treatment-naïve surgical specimens and 221 neoadjuvant-treated patients	Immune gene array, H&E/IHC, HEV-focused IHC, biomolecular analyses, and CODEX cellular-neighborhood analysis	Ectopic lymphoid structures; HEV proportion; TLS density and maturity; invasive-front cellular neighborhoods	High TLS density and maturity predicted RFS and neoadjuvant response in LARC; cellular-neighborhood coordination contextualizes TLS biology	CODEX cellular-neighborhood data are contextual rather than TLS-specific; rectal and colon cohorts should not be pooled without calibration
Nasopharyngeal carcinoma	Liu et al.; Li et al. (11, 48)	77 biopsy/blood samples; 343,829 cells; spatial transcriptomes from 15 tumors; Stereo-seq n=3, Visium n=12; microdissection cohort n=189	Multiplex IHC, scRNA-seq, immune-receptor profiling, Stereo-seq, Visium FFPE, and microdissection RNA-seq	CXCL13+ CAFs; CXCL13+ CD8/CD4 T cells; Tfh-like cells; B/plasma-cell maturation; TLS proximity to tumor aggregates	TLS-related cell signatures correlate with prognosis and PD-1 blockade response	EBV-associated and disease-specific NPC biology may limit transferability
Head-and-neck SCC	Li et al. (56)	HNSCC samples stratified by TLS status; exact cohort size not reported in this table	scRNA-seq, scTCR-seq/scBCR-seq, pathology/IHC	Mature TLSs; progenitor-exhausted CD4+ T cells with Tfh-like features; B-cell, plasma-cell, DC-LAMP+ DC, and CD8+ T-cell activation	Mature TLSs evoke local T- and B-cell responses through helper-state programs	Exact cohort size and cross-cohort validation requirements remain to be confirmed
Cervical cancer	Huang et al.; Gorvel et al. (17, 67)	Cervical cancer cohorts; sample size not reported in accessible metadata checked here	Multi-omics profiling and pathology/immune-contexture analysis; exact platform details not confirmed from accessible metadata checked here	Immunosuppressive plasma-cell subset; macrophage activation states; immune-checkpoint expression in TLS context	TLS context predicts outcome, but mature-looking humoral niches may be immunosuppressive	Plasma-cell abundance should not be assumed uniformly favorable; platform details should be checked against the final full text
PDAC	Amisaki et al.; Ahmed et al. (50, 51)	Human PDAC cohorts; PDAC mouse models; engineered recombinant IL-33 intervention	Bulk mRNA correlation, IHC, immunofluorescence, mouse PDAC models, and single-cell transcriptomic analysis of ILC2s	IL-33+ cells; lymphoneogenic ILC2s; LTβR+ myeloid organizer cells; TLS density and maturation states	IL-33-activated ILC2s induce TLSs; recombinant IL-33 expands intratumoral ILC2s and TLSs and enhances antitumor activity in PDAC mice	Preclinical induction strategy; microbiota dependence and type-2 inflammation risk remain unresolved
High-grade serous ovarian cancer	MacFawn et al.; Varghese et al. (39, 68)	HGSOC spatial/immunophenotyping studies and ovarian-cancer TLS review; exact sample sizes not reported in accessible metadata checked here	GeoMx digital spatial transcriptomic profiling, multispectral immunofluorescence/IHC, and spectral flow cytometry (39)	Site- and stroma-governed TLS activity	TLS activity is shaped by anatomical site and may help explain variable or weak ICI response	Sampling site is critical; primary, metastatic, and peritoneal lesions may differ; not all B-cell states are favorable
Glioma/glioblastoma	van Hooren et al.; Lee-Chang et al. (60, 69)	Human glioma specimens and GL261/CT-2A murine glioma models	Histology/IHC, immune profiling, αCD40 intervention, and 4-1BBL/CD40-based immune-activation models	TLS-like clusters defined as compact CD45+ aggregates with dense B220+ B-cell core; B-cell activation features	αCD40 increased TLS formation but impaired checkpoint blockade responses in glioma models; 4-1BBL+ B-cell activation can elicit antitumor immunity	CNS anatomy and model-specific immune context make TLS induction especially risky to generalize
Pan-cancer atlas	Li et al.; Cho et al. (33, 70)	Two pan-cancer studies; 2026 atlas: 12 cancer types	Spatial transcriptomics with ultrahigh-plex validation	TLS maturation, niche remodeling, and distance gradients	Tissue-dependent programs; maturation-aware score stratified disease and treatment contexts	Cross-tumor use requires calibration

CAF, cancer-associated fibroblast; CODEX, co-detection by indexing; DC, dendritic cell; HEV, high endothelial venule; HGSOC, high-grade serous ovarian cancer; ICI, immune checkpoint inhibitor; IHC, immunohistochemistry; LARC, locally advanced rectal cancer; NSCLC, non-small-cell lung cancer; PDAC, pancreatic ductal adenocarcinoma; TLS, tertiary lymphoid structure.

Platform, sample/cohort, TLS readout, endpoint, and limitation are separated to avoid treating heterogeneous evidence streams as interchangeable. “Not reported” indicates that the specific item could not be confirmed from the accessible original article text/metadata checked during this revision or was not applicable to the study type. Reader guide: compare context, platform, TLS definition, inference, and limitations across columns; match the platform to the biological question and error profile.

Whole-transcriptome, spot-based spatial transcriptomics and high-plex protein imaging have distinct failure modes. The former offers broad molecular discovery but is sensitive to spot mixing, variable gene capture, RNA quality, batch effects, and model-dependent deconvolution. The latter can resolve TLS architecture, HEV association, and B/T-cell zoning but depends on antibody and panel performance, staining consistency, image registration, tissue integrity, control of autofluorescence or signal spillover, and cell segmentation. In prospective translational workflows, transcriptomics offers molecular breadth but requires computational and cross-platform harmonization, whereas an analytically validated, pathologist-reviewed imaging panel can retain interpretable morphology but is limited to the targets included in the panel. When both modalities are used, they should be treated as complementary assays with prespecified specimen, quality-control, segmentation, scoring, and failure criteria, rather than as interchangeable reference standards (27–29, 36).

### Single-cell integration and computational pathology

The most informative contemporary TLS studies triangulate three layers: histopathology for structure, spatial omics for tissue context, and single-cell transcriptomics, often paired with T-cell and B-cell receptor profiling and single-cell chromatin-accessibility profiling—for state annotation. This arrangement enables cell states first described in dissociated material to be remapped onto intact tissue. It also demands caution: dissociation may enrich resilient immune subsets while under-representing fragile stromal or endothelial compartments, so some TLS circuits appear deceptively simpler in single-cell data than on tissue sections (28, 41).

Computational pathology has become the fourth layer. Automated TLS detection, follicle segmentation, HEV counting, neighborhood analysis, and joint modeling with clinical endpoints are now feasible at cohort scale. Deep-learning tools such as HookNet-TLS, a multi-resolution convolutional architecture that quantifies lymphoid aggregates and germinal centers in routine H&E slides (42, 43), and TLS-PAT, which classifies colorectal-cancer TLS maturity with pathologist-level concordance (44), have begun standardizing what pathologists have traditionally identified manually. This matters because biomarker translation cannot advance if every study uses idiosyncratic TLS definitions, inconsistent distance thresholds for “intratumoral” positioning, or incompatible maturity rules (9, 10, 42–44). Orthogonal validation through multiplex immunofluorescence, RNAscope or FISH, flow cytometry, and functional assays remains essential before any spatially derived signature enters clinical decision-making, as emphasized by the validation and translation panels of **Figure 2**. Accordingly, the bridge from platform selection to clinical translation is to identify the TLS module relevant to the clinical question and measure it with the simplest validated assay that preserves the required spatial information.

For TLS research, these emerging platforms could resolve B/T-cell zoning, chemokine-producing stromal niches, and TLS–tumor interfaces, while distinguishing follicular cores from surrounding suppressive or metabolically altered niches. Histology and spatial proteomics remain necessary to validate maturity and HEV coupling. Cui and colleagues review the relationship between spatial cellular communities, including TLSs, and therapeutic response, supporting integrated profiling with functional and clinical validation (45).

## Cellular and molecular programs governing TLS initiation and maturation

Once TLSs are analyzed as neighborhoods rather than counts, several recurring modules become visible. Productive TLSs require coordinated stromal licensing, vascular specialization, lymphocyte recruitment, and local differentiation. They stall when any of these modules is missing, subverted, or metabolically disabled. These four modules, together with their downstream functional outputs, are systematically summarized in **Figure 3**, which serves as the conceptual anchor for the following subsections.

**Figure 3:**
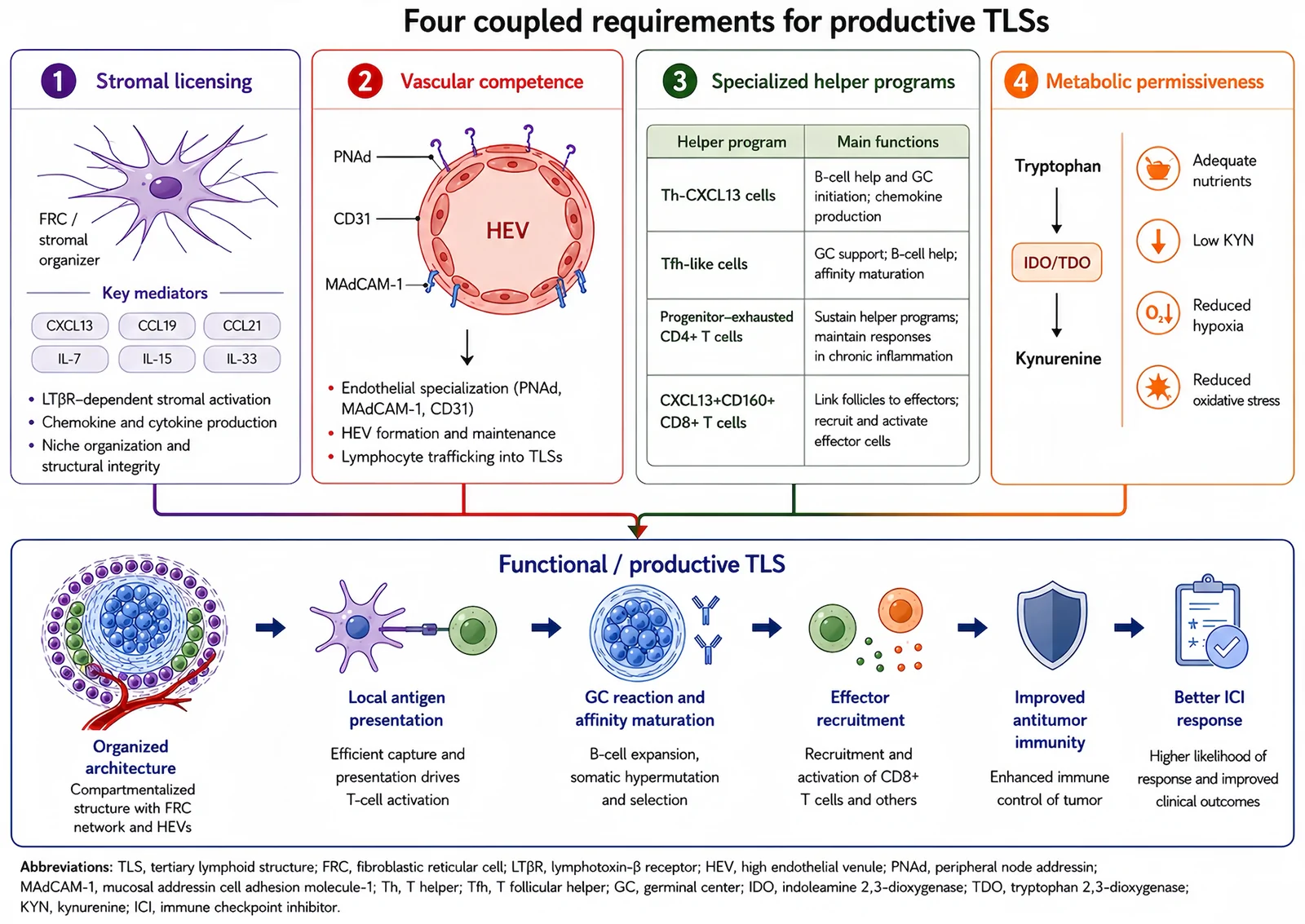
Four coupled requirements for productive TLSs. The schematic summarizes four non-overlapping mechanistic modules required for productive TLS function: stromal licensing through LTβR-dependent organizer-cell and chemokine programs; vascular competence through HEV formation, PNAd/CD31/MAdCAM-1 expression, endothelial specialization, and lymphocyte trafficking; specialized helper programs involving Th-CXCL13, Tfh-like, progenitor-exhausted CD4+ T-cell, and CXCL13+CD160+CD8+ T-cell states; and metabolic permissiveness involving adequate nutrients, constrained IDO/TDO-driven tryptophan catabolism, low kynurenine, and reduced hypoxic or oxidative stress. The lower panel links these modules to organized architecture, antigen presentation, germinal-center activity, effector recruitment, improved antitumor immunity, and better immune-checkpoint-inhibitor response. LTβR, lymphotoxin-β receptor; HEV, high endothelial venule; PNAd, peripheral node addressin; IDO, indoleamine 2,3-dioxygenase; TDO, tryptophan 2,3-dioxygenase; KYN, kynurenine; ICI, immune checkpoint inhibitor. The numbered, color-coded panels identify modules 1-4, and arrows show their convergence on a productive TLS and the sequence of downstream outcomes. A plus sign (+) denotes marker-positive cells. CCL19/CCL21, C-C motif chemokine ligands 19/21; CD4/CD8/CD31/CD160, cluster of differentiation 4/8/31/160; CXCL13, C-X-C motif chemokine ligand 13; FRC, fibroblastic reticular cell; GC, germinal center; IL-7/IL-15/IL-33, interleukins 7/15/33; MAdCAM-1, mucosal addressin cell adhesion molecule 1; Th, T helper; Tfh, T follicular helper cell; TLS, tertiary lymphoid structure.

### Stromal licensing and chemokine circuitry

Fibroblastic and organizer-like stromal cells occupy the top of the hierarchy. Foundational work on chronic inflammation established that lymphotoxin signaling, local chemokine production, and stromal scaffolding are prerequisites for tertiary lymphoid organogenesis, and tumor studies have progressively confirmed this logic (8, 46, 47). In practice, LTα1β2 binding to LTβR on fibroblastic reticular cells initiates an organizer program. This induces CXCL13, CCL19, and CCL21, while IL-7, IL-15, and IL-33 reinforce TLS survival and maturation (**Figure 3**, module 1).

Recent tumor-focused spatial studies have mapped these signals to concrete cellular sources. In nasopharyngeal carcinoma, CXCL13^+^ CAF-like stromal populations and specialized T-cell subsets collectively form the backbone of TLS niches (11, 48). In gastric and pan-cancer datasets, stromal and immune chemokine modules segregate with maturation state rather than simply with lymphocyte abundance (20, 32, 49). These observations argue against a naïve “more inflammation, more TLS” model; the decisive variable is whether inflammation is organized around a stromal scaffold capable of maintaining B- and T-cell zoning.

An important recent development has extended this framework upstream of classical LTβR signaling. Amisaki and colleagues demonstrated that interleukin-33 (IL-33), the archetypal alarmin released from damaged epithelium, activates group 2 innate lymphoid cells (ILC2s) that express lymphotoxin and subsequently engage LTβR^+^ myeloid organizer cells to initiate lymphoneogenesis in murine pancreatic ductal adenocarcinoma (PDAC) (50). Lymphoneogenic ILC2s trafficked from the gut to PDAC lesions, were modulated by the gut microbiota, and could be pharmacologically expanded with an engineered recombinant IL-33 protein that enhanced antitumor immunity. This IL-33–ILC2–LTβR axis provides a mechanistically tractable entry point for inducing TLSs in tumors that historically lack them (50, 51).

### HEVs, endothelial remodeling, and vascular competence

HEVs are often treated as accompanying features of mature TLSs, but spatial evidence suggests they play a far more active role. These specialized post-capillary venules enable L-selectin–mediated lymphocyte entry and connect local inflammation to a wider trafficking network (52–54). Vascular competence therefore depends on both HEV formation and function, including the distribution of PNAd, MAdCAM-1, and CD31 and the efficiency of transendothelial lymphocyte migration (**Figure 3**, module 2).

Vascular normalization experiments anticipated this concept. LIGHT-mediated vascular targeting drove the *de novo* formation of intratumoral lymphoid structures, normalized vessel architecture, and improved responses to ICI therapy in otherwise resistant murine tumors (22). Subsequent clinical-pathology studies have extended this principle to human cancers: HEV abundance within TLSs predicts favorable immune contexture and survival in colorectal cancer, and NSCLC treated with neoadjuvant immunochemotherapy (16, 38). A therapeutically useful TLS score should therefore interrogate not only whether a lymphoid aggregate exists but whether it is vascularly competent.

Spatial single-cell profiling has recently refined this concept further. Matsumoto and colleagues identified ID1-high endothelial cells within TLSs as candidate predictors of durable ICI response in NSCLC, supporting the idea that endothelial heterogeneity within HEV-containing TLSs, rather than HEV presence alone, may influence functional outcome (41). Complementary work linking endothelial Notch-signaling loss to spontaneous TLS formation in non-tumor inflammatory settings (55) suggests that endothelial programs can influence lymphoid assembly, although direct therapeutic tuning in cancer requires additional validation.

### T-cell help, B-cell selection, and local differentiation

B-cell biology brought TLSs into the immunotherapy mainstream, yet B cells do not act in isolation. Landmark melanoma and sarcoma cohorts linked B cells and TLSs to clinical benefit after ICI therapy, but the signal that unified these datasets was organized local immunity rather than B-cell abundance alone (4–6). As summarized in **Figure 3**, module 3, three specialized T-cell programs recur across solid tumors: Th-CXCL13 cells that sustain B-cell help and germinal-center reactions; progenitor-exhausted CD4^+^ T cells that bridge follicular help and cytotoxic output; and CXCL13^+^CD160^+^ CD8^+^ T cells that couple follicles to effector function.

Spatial and single-cell analyses have refined the T-cell half of the circuit. In nasopharyngeal carcinoma, a PD-1^+^CXCR5^-^ helper subset co-expressing CXCL13 drove B-cell recruitment and TLS assembly (48). In head-and-neck squamous cell carcinoma, mature TLSs were associated with progenitor-exhausted CD4^+^ T cells capable of sustaining intratumoral T- and B-cell responses—mechanistically provocative because it places a canonically exhausted state within a helpful rather than purely dysfunctional niche (56). More recent evidence from renal cell carcinoma shows that TLSs harbor tumor-specific T-cell clones with stem-like progenitor features, while macrophage neighborhoods with an inferred immunosuppressive phenotype at tumor margins may counterbalance this response (57). In advanced gastric cancer, intratumoral CXCL13^+^CD160^+^CD8^+^ T cells were implicated in TLS formation and enhanced immunotherapy activity (58). Although these studies do not converge on a single canonical helper identity, they share a principle: TLS competence depends on specialized CXCL13-linked T-cell programs that bridge cytotoxic and follicular immunity.

Local B-cell differentiation then shapes the functional output. GC reactions, antibody class-switching, and plasma-cell emergence are central features of mature TLSs, yet terminal differentiation is not uniformly beneficial. In nasopharyngeal carcinoma, plasma cells interspersed with tumor aggregates in a pattern consistent with local antitumor activity (11). In cervical cancer, by contrast, multi-omics profiling identified an immunosuppressive plasma-cell subset within TLSs associated with a less favorable program (17). A CD20^+^CD22^+^ADAM28^+^ memory B-cell population has also been reported as enriched in ICI responders (59). The lesson is not that plasma cells are intrinsically good or bad; rather, lineage output must be interpreted alongside phenotype and position.

### Circuits of arrested or suppressive maturation

Perhaps the most important conceptual correction supplied by spatial studies is that a TLS can fail even after it forms. Tregs constitute one well-characterized failure mode. Experimental lung-cancer work demonstrated that Tregs infiltrating tumor-associated TLSs suppress antitumor T-cell responses (18), and subsequent human NSCLC data linked TLS-infiltrating Tregs to worse outcomes (19).

Tumor metabolism represents a second, biologically distinct failure mode. In HCC, Tang and colleagues resolved two immature TLS trajectories at near-single-cell spatial resolution and linked the unfavorable (“deviating”) trajectory to tumor-derived tryptophan catabolism; pharmacologic inhibition of this pathway rescued maturation and synergized with anti–PD-1 therapy in murine models (12). This model is particularly compelling because it connects a diffusible tumor metabolite to a highly local architectural defect: the structure is physically present but does not complete productive maturation. The metabolic-permissiveness module therefore encompasses adequate nutrient availability, constrained IDO/TDO-driven tryptophan degradation, low kynurenine accumulation, and a supportive metabolic microenvironment more broadly (**Figure 3**, module 4). Taken together, these suppressive circuits help explain why apparently conflicting clinical reports often reflect biological heterogeneity and measurement error rather than statistical noise alone. Mature, HEV-coupled, B-cell- and helper-T-cell–supported structures are simply not equivalent to Treg-rich or metabolically derailed aggregates (9, 22, 23).

### TLS presence, TLS maturation, and TLS-mediated antitumor function are separable states

A central interpretive point is that TLS presence, TLS maturation, and TLS-mediated antitumor function should be treated as three related but non-equivalent states (**Figure 4**). Presence refers to the histological recognition of a lymphoid aggregate. Maturation refers to architectural features such as B/T zoning, FDC networks, germinal-center formation, plasma-cell differentiation, and HEV development. Antitumor function refers to whether the niche actually supports antigen presentation, local B-cell selection, helper-cell activity, effector-cell recruitment, and clinically meaningful tumor control.

**Figure 4:**
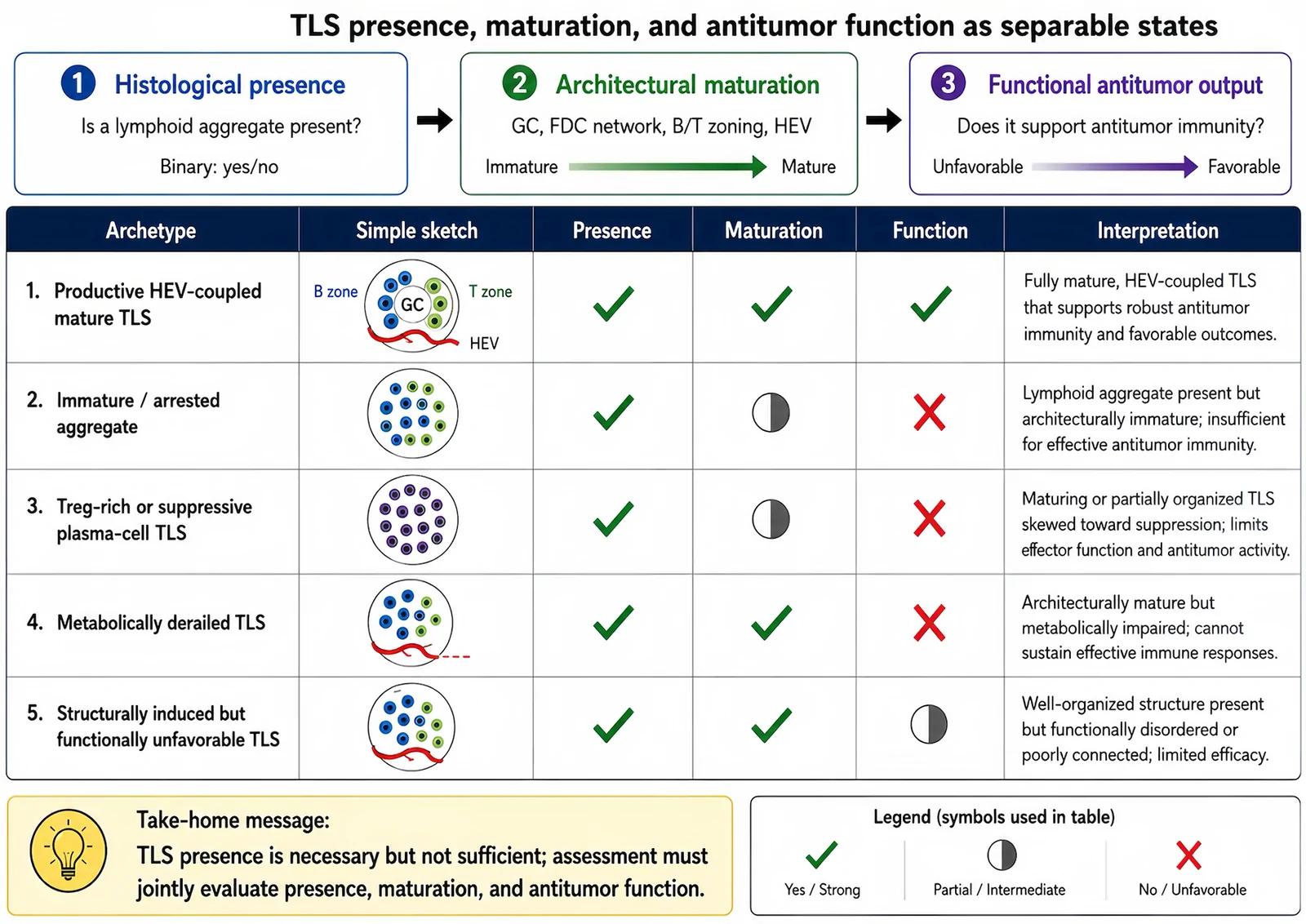
TLS presence, maturation, and antitumor function as separable states. The figure separates three related but non-equivalent interpretation levels: histological presence, architectural maturation, and functional antitumor output. The matrix compares productive HEV-coupled mature TLSs, immature/arrested aggregates, Treg-rich or suppressive plasma-cell TLSs, metabolically derailed TLSs, and structurally induced but functionally unfavorable TLSs. Check, partial, and cross symbols indicate whether each archetype is present, mature, and functionally favorable. The take-home message is that TLS presence is necessary but not sufficient; maturity and antitumor function must be evaluated together. B, B cell; FDC, follicular dendritic cell; GC, germinal center; HEV, high endothelial venule; T, T cell; TLS, tertiary lymphoid structure; Treg, regulatory T cell. Symbol key: green check mark, yes/strong; half-filled circle, partial/intermediate; red cross, no/unfavorable. Black arrows connect the three assessment levels; the green and purple gradient arrows indicate immature-to-mature and unfavorable-to-favorable continua, respectively.

These states can be uncoupled. An aggregate may be present but immature when it lacks FDC organization, HEV coupling, or adequate chemokine scaffolding. A mature-looking structure may still be functionally compromised if Tregs, suppressive plasma-cell programs, inhibitory myeloid states, or IDO/TDO-driven tryptophan catabolism dominate the local niche (12, 17–19). Conversely, a small or only partially mature TLS-like aggregate may be biologically important when it is intratumoral, HEV-coupled, and enriched for CXCL13-linked helper programs.

This tripartite distinction also clarifies therapeutic risk. Strategies that induce lymphoid architecture without correcting the surrounding suppressive context may increase TLS counts without improving immunity. The glioma CD40 example, in which agonistic CD40 therapy generated TLSs yet impaired checkpoint-blockade response, is therefore not an outlier but a warning that structural induction must be evaluated alongside maturity and functional orientation (60).

## What recent spatial studies reveal across tumor types

Across diseases, spatial studies reveal recurring TLS modules with variable, disease-specific wiring. The pertinent question is no longer whether TLSs exist, but which TLS variants dominate a given tumor ecosystem, which platform was used to define them, and which module is most limiting for productive immunity. **Table 1** therefore summarizes representative studies as an evidence map, separating sample context, named platforms, TLS readouts, clinical endpoints, and key limitations.

### Lung cancer and melanoma

Lung cancer remains the reference system because both foundational histology and subsequent mechanistic work have been unusually strong in this disease. H&E- and IHC-based pathology first associated intratumoral lymphoid structures with durable survival in NSCLC (1), and subsequent immunohistochemical and immune-contexture analyses showed that dendritic cells and B cells within TLSs shape a Th1-skewed cytotoxic contexture (13, 14). Meta-analytic data now place the overall-survival hazard ratio for TLS-high NSCLC at approximately 0.48 (95% CI 0.35-0.66) (61), providing a robust aggregate effect size on which composite scoring can be benchmarked. More recently, spatial transcriptomics and multiplex imaging showed that CCR7-positive mature dendritic cells accumulate in human TLSs and that type 1 conventional dendritic cells can orchestrate TLS development and maintenance through CCL19 stromal hubs and local antigen presentation (62).

Modern datasets add topography, maturity, and endothelial state. Digital pathology and spatial-distribution analyses of NSCLC demonstrate that TLS location across tumor regions materially influences clinical meaning, while HEV-focused IHC in neoadjuvant immunochemotherapy cohorts links HEV-rich TLSs to treatment response (15, 38). A TLS-derived gene-signature study using RNA-seq data from ORIENT-11, OAK, POPLAR, and TCGA-LUAD identified TLS signature 3 as a predictor of first-line chemoimmunotherapy response and a complement to PD-L1 testing, while also emphasizing that transcriptomic signatures do not directly resolve TLS spatial maturity (63). Single-cell spatial identification of ID1-high endothelial cells further refines which TLS-associated endothelial states may translate into durable ICI benefit (41). In melanoma, the message is similar but was articulated through pathology and immune-profiling studies of checkpoint blockade: organized B-cell-rich TLS niches, rather than diffuse lymphocyte accumulation, were linked to benefit (5, 64).

### Hepatobiliary and gastric cancers

Hepatocellular and gastric cancers have emerged as particularly informative models for spatial TLS biology because they showcase both successful and arrested maturation. Early HCC histopathology linked intratumoral TLSs to better prognosis (65), but newer spatial work has explained why some TLSs remain beneficial while others deviate (12).

In HCC specifically, near-cellular spatial transcriptomics connected conforming TLSs to antitumor immunity and anti–PD-1 responsiveness, whereas deviating TLSs associated with tumor tryptophan catabolism (12). In gastric cancer, integrated single-cell and spatial transcriptomic profiling of 30 gastric cancer specimens—25 profiled by single-cell RNA sequencing and five by 10x Visium spatial transcriptomics—stratified tumors by TLS spatial localization and identified CXCL13-linked helper/cytotoxic T-cell programs, ACKR1+ HEV-related interactions, and a single-cell/spatial TLS signature with prognostic and immunotherapy-response relevance (32). Cross-cancer single-cell and spatial transcriptomic analysis of colorectal and gastric tumors then demonstrated that shared chemokine interactions coexist with cancer-type-specific cellular wiring (49). In locally advanced rectal cancer, H&E/IHC-based assessment of TLS density and maturity predicted recurrence-free survival in treatment-naïve surgical specimens (HR 0.2985, 95% CI 0.1894–0.4706) and TLS positivity in pre-treatment specimens was associated with improved neoadjuvant-treatment response (66), extending the colorectal-TLS paradigm into treatment contexts.

These gastrointestinal datasets are useful precisely because they reject an over-simple narrative. They show that TLS maturation is not a monotonic process driven by lymphocyte quantity. Local chemokine topology, endothelial context, and tumor metabolism jointly determine whether the structure becomes a site of immune education or a partially organized bystander.

### Nasopharyngeal, head-and-neck, and cervical cancers

Head-and-neck malignancies illustrate the diversity of TLS-supporting cell states. In nasopharyngeal carcinoma, single-cell and spatial transcriptomic studies mapped CXCL13^+^ CAFs, Th-CXCL13 cells, stem-like CXCL13^+^CD8^+^ T cells, B cells, and T-follicular-helper cells into a coordinated TLS ecosystem that also directly intersects with tumor-cell aggregates (11, 48). Head-and-neck squamous cell carcinoma broadens this concept by using single-cell and spatial transcriptomic analyses to implicate progenitor-exhausted CD4^+^ T cells in mature TLS-associated immunity (56), placing a classically exhaustion-defined state within a helpful rather than purely dysfunctional niche. The study suggests that local helper competence can be preserved within partially exhausted programs when the surrounding architecture is mature.

Cervical cancer, by contrast, provides a cautionary example. Multi-omics profiling with spatially resolved immune-context analysis identified an immunosuppressive plasma-cell subset within TLSs, supporting the principle that local antibody production can either enhance or blunt antitumor immunity depending on the differentiation pathway and tissue context (17). Parallel pathology and immune-profiling work has further implicated macrophage activation states and immune-checkpoint expression within cervical TLSs as independent outcome determinants (67).

### Historically cold tumors: pancreatic, ovarian, and glioma

The translational frontier has shifted toward tumors in which TLSs are rare, anatomically constrained, or functionally ambiguous—PDAC, high-grade serous ovarian cancer, and glioma. In PDAC, the IL-33–ILC2–LTβR axis described by Amisaki and colleagues represents a mechanistically druggable lymphoneogenic pathway, with engineered IL-33 expanding intratumoral lymphoneogenic ILC2s and TLSs and enhancing antitumor activity in preclinical PDAC models (50, 51). In high-grade serous ovarian cancer, recent spatial and immunophenotyping studies indicate that TLS activity is influenced by anatomical site, stromal context, and cellular interactions (39, 68). These features help explain why TLS assessment in gynecological malignancies should account for sampling site and B-cell functional state rather than simply recording the presence of lymphoid aggregates.

In glioma, the blood–brain barrier and neural-stroma environment constrain TLS formation, but emerging evidence suggests that agonistic CD40 or 4-1BBL-based strategies can induce lymphoid-like structures (60, 69). A critical cautionary finding is that agonistic CD40 therapy in murine glioma generates TLSs yet paradoxically impairs the response to ICI therapy (60), demonstrating that structural induction alone does not guarantee functional benefit. The cold-tumor literature thus converges on a shared principle: engineering TLSs requires simultaneous attention to the immunosuppressive context in which they form.

### Pan-cancer lessons

Pan-cancer spatial transcriptomic integration suggests that TLSs are most accurately understood as a modular system. The recurrent modules, including stromal licensing, HEV formation, CXCL13-centered helper programs, and B-cell activation, differ in their relative contribution across tissues, treatment contexts, and tumor ecologies (9, 10, 20, 33). This conclusion carries two immediate implications. First, biomarker models trained in one tumor type may fail when exported without recalibration. Second, therapies aimed at inducing TLSs should target the missing module in a given tumor rather than assume that every cancer lacks the same element. A 2026 pan-cancer spatial atlas subsequently linked TLS maturation to coordinated remodeling of niche cell populations and derived a maturation-aware composite score that outperformed conventional TLS metrics across cancer and treatment contexts (70). These cross-tumor patterns provide the biological bridge from platform-specific observations to the composite biomarker framework considered next.

## TLSs as biomarkers in the spatial era

The TLS biomarker literature initially treated TLSs as countable pathology objects. This approach was productive for discovery but is no longer sufficient for clinical translation. Mature TLSs predict ICI benefit independently of PD-L1 across several solid tumors (37), yet effect size varies considerably across cohorts, and density alone rarely explains the variance (23, 71). A formal meta-analysis of 19 ICI trials (n = 1,752) estimated a response-rate odds ratio of 2.99 (95% CI 2.14–4.18; P < 0.001) and a PFS hazard ratio of 0.75 (95% CI 0.63–0.88; P < 0.001) for TLS-high patients (23). Although these aggregate figures are substantial, they represent a ceiling beneath which heterogeneity in TLS state, rather than density alone, accounts for much of the residual unexplained variance.

The novelty of the composite framework proposed here is not that density, maturity, location, HEV coupling, or composition are individually new variables. Rather, the novelty lies in combining them into a staged, module-matched clinical workflow that ties each dimension to feasible assays and to a therapeutic interpretation. Digital pathology first detects TLS structure and spatial position on H&E or whole-slide images; targeted multiplexed imaging then confirms maturity, B/T zoning, FDC networks, and HEV coupling; and compact RNA, protein, or metabolic panels add stromal, helper-cell, and metabolic information only where it changes interpretation (**Figure 5**; **Table 2**).

**Figure 5:**
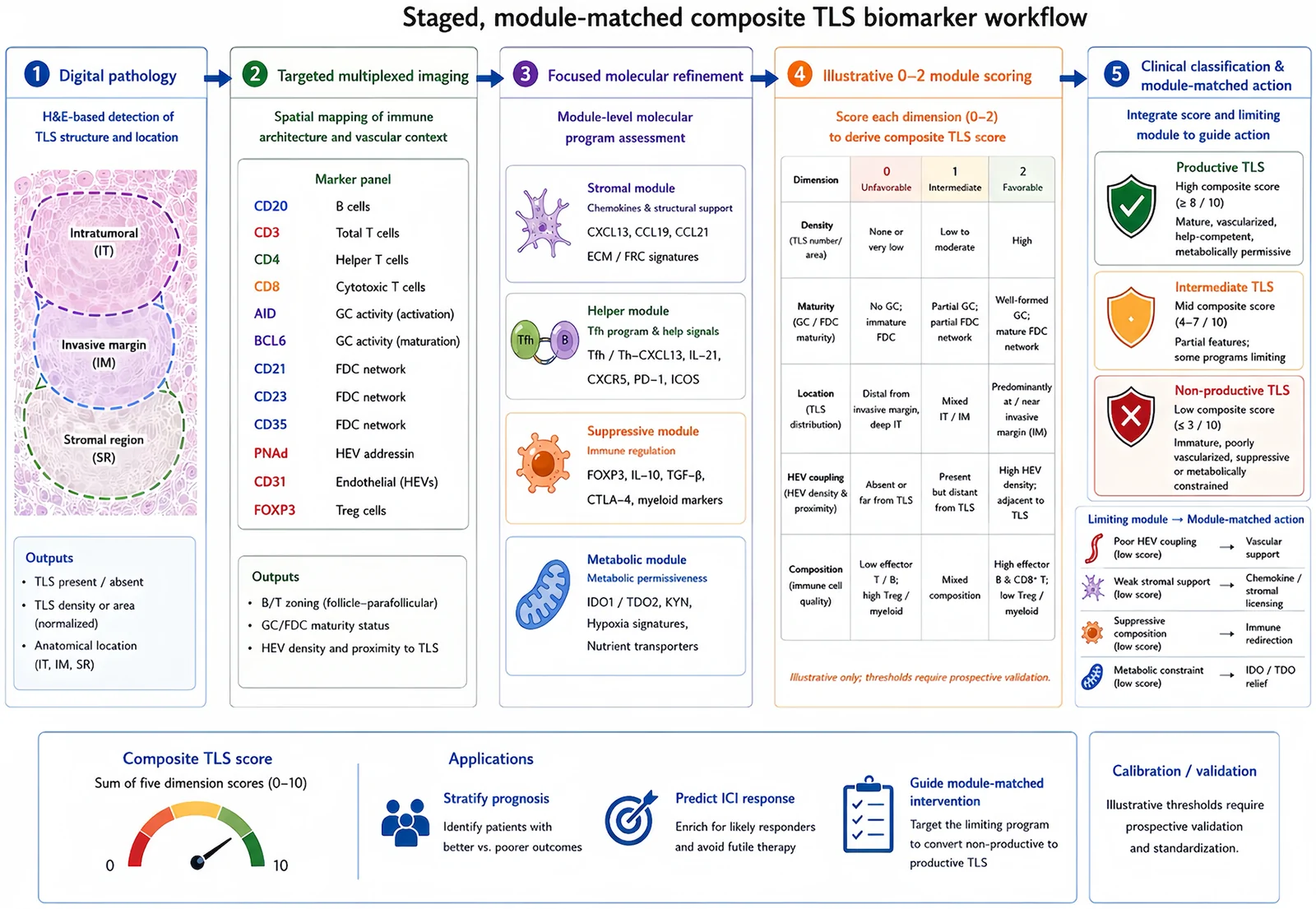
Staged, module-matched composite TLS biomarker workflow. The workflow operationalizes the composite TLS framework as a clinical testing sequence: digital pathology detects TLS structure, density, and anatomical location; targeted multiplexed imaging confirms B/T zoning, germinal-center/FDC maturity, and HEV coupling; focused RNA, protein, or metabolic panels refine stromal, helper-cell, suppressive-cell, and metabolic modules; an illustrative 0–2 scoring table integrates density, maturity, location, HEV coupling, and composition; and the resulting productive, intermediate, or non-productive TLS classification is linked to module-matched therapeutic logic. The scoring thresholds are illustrative and require prospective calibration and validation before clinical use. AID, activation-induced cytidine deaminase; B/T, B-cell/T-cell; BCL6, B-cell lymphoma 6; CCL19/CCL21, C-C motif chemokine ligands 19/21; CD3/CD4/CD8/CD20/CD21/CD23/CD31/CD35, cluster of differentiation 3/4/8/20/21/23/31/35; CTLA-4, cytotoxic T-lymphocyte-associated protein 4; CXCL13, C-X-C motif chemokine ligand 13; CXCR5, C-X-C motif chemokine receptor 5; ECM, extracellular matrix; FDC, follicular dendritic cell; FOXP3, forkhead box P3; FRC, fibroblastic reticular cell; GC, germinal center; H&E, hematoxylin and eosin; HEV, high endothelial venule; ICOS, inducible T-cell co-stimulator; ICI, immune checkpoint inhibitor; IDO, indoleamine 2,3-dioxygenase; IDO1, indoleamine 2,3-dioxygenase 1; IL-10/IL-21, interleukins 10/21; IM, invasive margin; IT, intratumoral; KYN, kynurenine; PD-1, programmed cell death protein 1; PNAd, peripheral node addressin; RNA, ribonucleic acid; SR, stromal region; TDO, tryptophan 2,3-dioxygenase; TDO2, tryptophan 2,3-dioxygenase 2; Tfh, T follicular helper cell; TGF-β, transforming growth factor beta; Th, T helper; TLS, tertiary lymphoid structure; Treg, regulatory T cell. Scores of 0, 1, and 2 indicate unfavorable, intermediate, and favorable values, respectively. Dashed contours identify intratumoral, invasive-margin, and stromal regions. In the classification panel, shield icons bearing a green check, orange diamond, and red cross denote productive, intermediate, and non-productive TLSs, respectively; arrows indicate workflow progression or linkage from a limiting module to its matched action. A plus sign (+) denotes marker-positive cells.

**Table 2:** Module-to-assay bridge and illustrative scoring logic for a composite TLS biomarker framework.

Module/dimension	Candidate readouts	Feasible assay(s)	Illustrative 0–2 score	Clinical interpretation/therapeutic logic	Common pitfall
Density and location	TLS number/area; intratumoral, invasive-margin, stromal, perivascular, peritumoral distribution; distance to tumor nests	H&E, WSI digital pathology, AI-assisted TLS detection, IHC confirmation	0 absent/rare or clearly distal; 1 present but peritumoral or spatially ambiguous; 2 intratumoral or invasive-margin and tumor-coupled	Initial screening and region selection for deeper profiling	Density alone collapses immature, mature, productive, and suppressive aggregates into one bin
Maturity/architecture	FDC network, germinal center, B/T zoning, plasma-cell fringe, follicle-like organization	IHC, mIF, CODEX, IMC, pathologist review	0 loose aggregate; 1 partial follicle or incomplete FDC/GC features; 2 GC-like or mature secondary follicle-like TLS	Separates transient inflammation from organized adaptive niches	Single B-cell marker staining is often misused as a maturity surrogate
Stromal licensing	CXCL13, CCL19, CCL21, LTβR-related programs, IL-7, IL-15, IL-33; organizer-like CAF/FRC states	RNAscope/FISH, targeted RNA panel, spatial transcriptomics, mIF with stromal markers	0 absent; 1 diffuse chemokine signal without organization; 2 spatially organized chemokine/stromal scaffold	Suggests need for stromal or chemokine induction when absent	CXCL13 source is ambiguous without spatial or cell-state annotation
Vascular competence/HEV coupling	PNAd, CD31, MAdCAM-1, HEV density, HEV–follicle distance, endothelial subtype markers such as ID1-high states, lymphocyte trafficking evidence	IHC, mIF, IMC, CODEX, spatial endothelial-state profiling	0 no HEV; 1 PNAd+ vessels sparse or distant; 2 perifollicular/intrafollicular HEVs with supportive endothelial features	Indicates whether lymphocyte entry/access is likely sufficient; may guide vascular-normalizing strategies	PNAd positivity alone does not prove functional trafficking or favorable endothelial state
Helper and cytotoxic programs	Tfh-like cells, Th-CXCL13 cells, progenitor-exhausted CD4+ T cells, CXCL13+CD160+ CD8+ T cells, dendritic-cell support	mIF/IMC/CODEX, scRNA-seq/snRNA-seq, TCR sequencing, targeted protein/RNA panels	0 absent or terminally dysfunctional; 1 mixed or poorly localized; 2 follicle-coupled helper/effector support	Guides vaccines, costimulatory agonists, or checkpoint combinations	Bulk T-cell scores often miss supportive helper states embedded inside TLSs
B-cell and plasma-cell output	GC B cells, memory B cells, BCR clonality, antibody-producing plasma cells, suppressive plasma-cell signatures	IHC/mIF, scRNA-seq, BCR sequencing, targeted RNA/protein panels	0 absent or suppressive-dominant; 1 mixed or ambiguous; 2 locally differentiated effector/humoral output without suppressive skew	Clarifies whether humoral differentiation supports or blunts antitumor immunity	Plasma-cell abundance is often assumed to be uniformly favorable
Metabolic permissiveness	IDO1, TDO2, kynurenine-related signatures, hypoxia, nutrient stress, acidic pH, tumor-adjacent metabolic gradients	Spatial transcriptomics, targeted RNA/protein panels, IHC, metabolite-informed assays where feasible	0 high suppressive metabolism; 1 partial constraint; 2 low catabolic/hypoxic pressure and supportive metabolic context	Identifies candidates for metabolic relief as TLS-maturation adjuvant	Metabolic readouts are rarely integrated with structural pathology
Specimen and treatment context	Core biopsy, resection, invasive margin, metastatic lesion, post-treatment specimen; baseline vs on-treatment status	Clinical annotation, pathology mapping, longitudinal sampling	Not a biological score; used for calibration and stratification	Prevents false comparison across specimen types and treatment time points	Pooling baseline and post-treatment tissues may obscure mechanism

BCR, B-cell receptor; CAF, cancer-associated fibroblast; CODEX, co-detection by indexing; FDC, follicular dendritic cell; FISH, fluorescence *in situ* hybridization; GC, germinal center; HEV, high endothelial venule; IHC, immunohistochemistry; IMC, imaging mass cytometry; mIF, multiplex immunofluorescence; PNAd, peripheral node addressin; scRNA-seq, single-cell RNA sequencing; snRNA-seq, single-nucleus RNA sequencing; Tfh, T-follicular helper; TLS, tertiary lymphoid structure; WSI, whole-slide imaging.

The 0–2 scoring examples are hypothetical and intended to illustrate operationalization; thresholds require prospective validation.

A next-generation TLS biomarker should therefore integrate at least four structural dimensions: density, maturity, anatomical location, and HEV coupling, with composition layered on top as a refining rather than replacement variable. Density provides coarse normalization, including the number or area of TLSs per unit tumor region. Maturity captures germinal centers, FDC networks, B/T zoning, and active B-cell maturation. Location distinguishes intratumoral, invasive-margin, stromal, perivascular, and peritumoral distributions. HEV coupling should not be reduced to PNAd positivity alone; it should also consider HEV–follicle distance, HEV density, CD31/PNAd co-localization, endothelial subtypes such as ID1-high states where feasible, and evidence of lymphocyte trafficking (16, 38, 41, 54). Composition—B-cell subsets, Tfh- and Th-like programs, CD8^+^ T cells, dendritic cells, Tregs, myeloid states, and plasma-cell polarization—refines interpretation once the structural backbone is confirmed, as summarized in **Table 2** (7, 9, 32).

For practical use, an illustrative 0–2 scoring scheme can make the workflow concrete while remaining explicitly hypothetical. For each dimension, 0 would indicate absence or a clearly unfavorable state; 1 would indicate partial or ambiguous support; and 2 would indicate a spatially organized, functionally favorable state. For example, HEV coupling could be scored 0 when HEVs are absent, 1 when PNAd-positive vessels are present but distant or sparse, and 2 when PNAd/CD31-positive HEVs are perifollicular or intrafollicular and accompanied by supportive endothelial features. A productive TLS profile would combine high structural score, mature architecture, intratumoral or invasive-margin coupling, HEV competence, supportive helper-cell states, and low suppressive/metabolic constraint. A non-productive profile would combine low maturity, poor vascular coupling, Treg- or suppressive plasma-cell enrichment, or high IDO/TDO/kynurenine-related metabolic pressure. These thresholds are offered only as an illustrative framework and require prospective calibration before clinical use.

Sampling strategy is an additional and underappreciated variable. Limited core biopsies may fail to capture peritumoral or invasive-margin TLSs, whereas resections sample a broader spatial field and are therefore not directly interchangeable with core biopsies. Invasive-margin sampling may preferentially capture margin-associated TLSs; TLS biology may vary across lesion sites; and treatment may induce or remodel TLSs relative to baseline. These design issues are simultaneously methodological and biological, which is why pathological scoring and spatial-omics validation should be co-developed rather than treated as independent pipelines. Accordingly, biomarker thresholds should be prespecified by specimen type, lesion site, sampled area, and treatment time point, and independently validated or recalibrated before use in a different context. In longitudinal studies, paired sampling should be anatomically matched whenever feasible because differences in lesion site, sampled area, or biopsy depth could confound attribution of a change in TLS state to treatment (10, 32, 39).

From a translational perspective, the most realistic near-term model is therefore a staged composite score: digital pathology defines structure and location, multiplexed imaging confirms HEV coupling and zoning, and focused transcriptomic, protein, or metabolic panels capture chemokine, helper-T-cell, and metabolic modules. When these modules are interrogated in concert, the resulting readouts provide a composite TLS score with three translational applications: patient prognostic stratification, prediction of ICI response, and guidance of therapeutic decisions (**Figure 5**; **Table 2**). The value of spatial omics therefore lies less in whole-transcriptome mapping for every patient and more in identifying which compact clinical panels should be measured prospectively (28, 29, 36). Clinical proof of concept for TLS-guided stratification already exists: in PEMBROSARC, TLS-positive soft-tissue-sarcoma patients had higher objective-response and six-month non-progression rates with pembrolizumab than historical all-comer cohorts (72). However, prospective validation of the full composite spatial-functional TLS framework remains future work. Once that score identifies the limiting module, the same result can guide a matched therapeutic strategy, which is the focus of the next section.

## Therapeutic engineering of TLSs

If TLSs were only correlated with outcome, they would remain useful but limited biomarkers. Spatial studies, in contrast, support a more ambitious view: TLSs are partially engineerable. The central challenge is that productive TLSs cannot be created by attracting lymphocytes in bulk. Stromal licensing, vascular specialization, and local differentiation must emerge in a coordinated sequence (3, 54, 73). The therapeutic opportunities surveyed in **Figure 6** can be grouped into three broad strategies: enhancing TLS formation and maturation via stromal, chemokine, and vascular cues; boosting helper-cell function and antigen presentation via vaccines, co-stimulatory agonists, checkpoint blockade, and adoptive cell therapy; and reprogramming the metabolic context to remove brakes on maturation.

**Figure 6:**
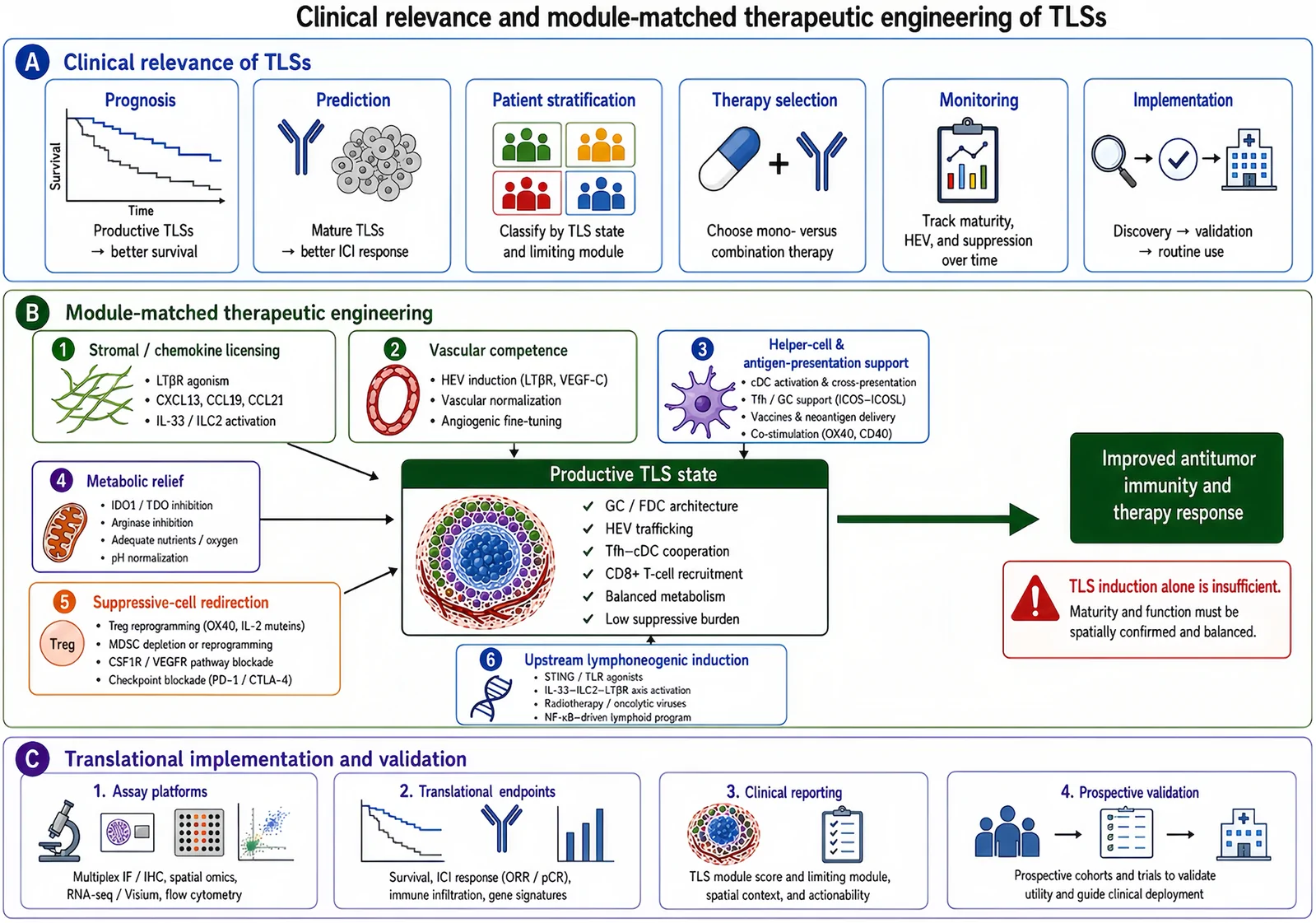
Clinical relevance and module-matched therapeutic engineering of TLSs. Section A summarizes clinical applications of spatially resolved TLS assessment, including prognostication, prediction of ICI response, patient stratification, therapy selection, monitoring, and implementation. Section B organizes therapeutic engineering by the limiting module: stromal/chemokine licensing, vascular competence, helper-cell and antigen-presentation support, metabolic relief, suppressive-cell redirection, and upstream lymphoneogenic induction in cold tumors. These interventions converge on a productive TLS state characterized by GC/FDC architecture, HEV trafficking, Tfh-cDC cooperation, CD8+ T-cell recruitment, balanced metabolism, and low suppressive burden. Section C outlines translational implementation and prospective validation. The caution box emphasizes that TLS induction alone is insufficient unless maturity and antitumor function are spatially confirmed. ICI, immune checkpoint inhibitor; LTβR, lymphotoxin-β receptor; STING, stimulator of interferon genes; TLR, Toll-like receptor; IDO1, indoleamine 2,3-dioxygenase 1; TDO, tryptophan 2,3-dioxygenase; IF, immunofluorescence; IHC, immunohistochemistry. CCL19, C-C motif chemokine ligand 19; CCL21, C-C motif chemokine ligand 21; CD8, cluster of differentiation 8; CD40, cluster of differentiation 40; cDC, conventional dendritic cell; CSF1R, colony-stimulating factor 1 receptor; CTLA-4, cytotoxic T-lymphocyte-associated protein 4; CXCL13, C-X-C motif chemokine ligand 13; FDC, follicular dendritic cell; GC, germinal center; HEV, high endothelial venule; ICOS, inducible T-cell co-stimulator; ICOSL, ICOS ligand; IL-2, interleukin 2; IL-33, interleukin 33; ILC2, group 2 innate lymphoid cell; MDSC, myeloid-derived suppressor cell; NF-κB, nuclear factor kappa B; ORR, objective response rate; OX40, tumor necrosis factor receptor superfamily member 4; pCR, pathological complete response; PD-1, programmed cell death protein 1; pH, measure of hydrogen-ion activity; RNA-seq, RNA sequencing; Tfh, T follicular helper cell; TLS, tertiary lymphoid structure; Treg, regulatory T cell; VEGF-C, vascular endothelial growth factor C; VEGFR, vascular endothelial growth factor receptor. Symbols: arrows indicate progression or convergence; the plus sign between therapy icons denotes combination therapy; a plus sign after CD8 denotes marker-positive cells; check marks identify the listed features of the productive TLS state; the red warning triangle identifies the caution that TLS induction alone is insufficient.

Preclinical vascular-targeting experiments made this point early. LIGHT-mediated vessel targeting normalized tumor vasculature, induced intratumoral lymphoid structures, and improved immunotherapy outcomes in resistant models (22). More recent work demonstrates that LTβR agonism can remodel the tumor microenvironment toward antitumor immunity (74), that loss of endothelial Notch signaling promotes spontaneous TLS formation (55), and that the IL-33–ILC2 axis provides an alarmin-triggered lymphoneogenic program amenable to recombinant-protein expansion (50). Agents targeting sympathetic nerves and hydrogel-based synthetic lymphoid scaffolds are additional emerging strategies that illustrate the breadth of engineering approaches under preclinical investigation (75, 76).

The stromal compartment offers a second intervention point. CAFs can orchestrate TLS assembly rather than merely wall it off, suggesting that selective stromal rewiring could support lymphoid organization when compatible endothelial and chemokine programs are concurrently present (8, 77). The metabolic axis constitutes a third intervention node: tryptophan-catabolism inhibitors, currently in clinical development for other indications, are now rationally indicated as TLS-maturation adjuvants in tumors where tryptophan-driven deviation has been documented (12).

However, induction is not automatically beneficial. Agonistic CD40 therapy in glioma generated TLSs but paradoxically impaired responses to checkpoint blockade (60), a cautionary example that structure alone does not guarantee productive immunity. Spatial multi-omics is uniquely positioned to improve intervention design by revealing which module is absent in a given tumor. A tumor with diffuse lymphocytes but poor HEV formation may require vascular programming; a tumor with organized follicles but suppressive plasma-cell or metabolic programs may require local redifferentiation or metabolic relief; and a fully cold tumor may require upstream lymphoneogenic induction, for example via IL-33, combined with downstream checkpoint therapy. This is a substantially more precise therapeutic logic than the indiscriminate pursuit of “more TLSs” (**Figure 6**; **Table 3**), as supported by prior mechanistic studies (12, 17, 54).

**Table 3:** Approaches to induce or reshape TLSs therapeutically, ordered from more established vascular/LTβR concepts to exploratory synthetic-scaffold and module-matched strategies.

Engineering strategy	Primary module targeted	Mechanistic lever	Representative evidence	Major caveat
Vascular targeting with LIGHT/TNFSF14	Vascular competence + lymphocyte entry	Combines vascular normalization with local immune organization and lymphocyte entry	Improved intratumoral lymphoid structure formation and ICI response in resistant tumors (22)	Preclinical proof of concept; transferability, dosing, and systemic effects remain unresolved
LTβR agonism	Stromal licensing	Activates lymphoid-organizing stromal programs and remodels the TME	Enhances antitumor immunity through TME modulation (74)	Requires compatible stromal and endothelial context to generate functional rather than incomplete structures
Endothelial Notch modulation	Vascular competence	Promotes endothelial states permissive for TLS formation and altered lymphocyte trafficking	Loss of endothelial Notch signaling triggered spontaneous TLS formation (55)	Likely systemic vascular effects and tissue-specific liabilities
IL-33/ILC2 axis activation	Upstream lymphoneogenic induction	Alarmin-triggered ILC2–LTβR engagement of myeloid organizer cells	Engineered recombinant IL-33 expanded intratumoral lymphoneogenic ILC2s/TLSs and enhanced antitumor activity in PDAC models (50, 51)	Microbiota dependence and type-2 inflammation require careful scheduling; clinical translation pending
Tryptophan-catabolism inhibition	Metabolic permissiveness	Relieves tumor-derived metabolic pressure on TLS maturation	IDO/TDO inhibition rescued deviating TLS maturation and synergized with anti–PD-1 in HCC (12)	Prior clinical failures of IDO1 inhibitors in unselected populations argue for TLS-guided selection
CAF or stromal rewiring	Stromal licensing/chemokine organization	Leverages fibroblast-dependent chemokine and organizer programs to support TLS assembly	CAF-dependent orchestration of TLSs demonstrated in tumor models (77)	Fibroblast states are heterogeneous; some support immune exclusion or fibrosis
Agonistic CD40 therapy	Immune activation/B-cell support	Strong systemic immune activation can trigger TLS-like structures	TLS induction was documented in glioma (60)	Structures formed did not improve—and in some settings impaired—checkpoint responses; caution in CNS tumors
Synthetic scaffolds/artificial TLSs	Structural scaffold + local immune organization	Self-assembled microparticle hydrogel scaffolds recapitulate lymphoid architecture for CAR-T augmentation	Proof-of-concept enhancement of CAR-T cell therapy against solid tumors (76)	Early preclinical; translation, scale-up, and regulatory path remain to be established
Combination therapy guided by TLS state	Module-matched bottleneck	Targets the missing module rather than boosting inflammation nonspecifically.	Supported by spatial studies separating productive from deviating TLS programs (12, 17, 54).	Requires spatially informed patient selection and longitudinal sampling.

CAR-T, chimeric antigen receptor T cell; TNFSF14, tumor necrosis factor superfamily member 14. Other abbreviations are defined in the main text.

## Future perspectives and outstanding challenges

Three technical challenges currently limit field progress more than the absence of biological markers. The first is definition drift. Papers continue to apply divergent criteria for TLS identification, follicular maturity, germinal-center assignment, and intratumoral versus peritumoral classification. Without a harmonized ontology, even robust biomarkers will remain difficult to reproduce (9, 10). Pathology-based deep-learning algorithms with pre-registered specifications offer one route to standardization; initiatives such as HookNet-TLS and TLS-PAT illustrate what cross-center reproducibility could look like when the definition itself is encoded in software (42, 44).

The second challenge is scale mismatch. Spatial transcriptomics remains expensive for large clinical cohorts, and multiplexed-imaging panels vary substantially across centers. A pragmatic translational pipeline will likely deploy whole-slide digital pathology for detection, a targeted imaging panel for architectural validation, and a compact RNA or protein panel for mechanistic augmentation rather than whole-transcriptome profiling in every case (29, 35, 36). A staged clinical pipeline—discovery and validation in well-annotated cohorts, assay standardization for reproducibility and scalability, prospective clinical validation, and regulatory approval with integration into clinical workflows—provides a credible implementation template (**Figure 6**).

Clinical implementation would require conversion of the discovery workflow into a locked assay with prespecified tissue requirements, marker panels, scoring rules, quality-control thresholds, software versions, and intended use. Multicenter analytical concordance and clinical validity in a defined treatment setting would be prerequisites for validation; prospective evidence that the result improves decision-making would be needed to establish clinical utility. Regulatory and reimbursement pathways would be jurisdiction- and indication-specific, and no established pathway for a composite TLS assay is implied here. In practice, we envisage routine H&E review as a triage step, with targeted imaging for TLS-positive or equivocal regions, structured pathology reporting, and additional molecular testing only when it could change the composite classification (29, 71, 78).

Cost and scalability should be assessed across the full testing pathway, including capital equipment, reagents, tissue consumption, specialist labor, image and data storage, bioinformatics, turnaround time, and repeat-testing rates. Whole-slide digital pathology offers a relatively scalable initial screening layer; depending on the intended use and local infrastructure, high-plex imaging or spatial transcriptomics could then be applied to selected cases or regions, equivocal specimens, or validation cohorts. Because local infrastructure and panel design strongly affect cost, universal per-sample price rankings should be avoided. Before broad adoption, a minimal deployable panel should be benchmarked against a pathology-only score and tested across centers with different resource levels (28, 29, 78).

The third challenge is causality. Contemporary human spatial datasets are rich enough to generate plausible models but insufficient to definitively establish which cell state initiates or aborts TLS maturation. Longitudinal sampling before and during therapy, matched T-cell receptor and B-cell receptor lineage tracing, and interventional studies designed around specific missing modules will all be required. The field also needs tighter integration of treatment context, because chemotherapy, radiotherapy, anti-angiogenic therapy, and checkpoint blockade each reshape TLS biology in distinct ways (4, 37, 38). AI-assisted integration of spatial multi-omics may accelerate hypothesis generation, although its application to TLS-directed treatment modeling remains conceptual (78).

A deeper conceptual challenge also deserves emphasis. TLSs should not be treated as miniature lymph nodes transplanted into tumors. They are contingent structures shaped by local fibroblasts, damaged vasculature, chronic antigen exposure, and distorted nutrient fields. That contingency is precisely why spatial multi-omics is valuable: it allows the structure to be studied in the landscape that gave rise to it. The next critical step should be prospective, treatment-specific validation of a minimal TLS panel rather than yet another generation of retrospective density-only scoring (79).

## Conclusions

TLS research has entered a phase in which morphology alone is no longer sufficient. Spatially detailed studies demonstrate that clinically meaningful TLSs are assembled through coordinated stromal, vascular, helper-T-cell, B-cell, and metabolic programs and can be derailed by regulatory or metabolic pressures even when the structure remains histologically visible. For biomarker development, this means density must be interpreted alongside maturity, location, composition, HEV coupling, sample context, and metabolic permissiveness. For therapy, it means successful TLS induction will require module-specific engineering rather than nonspecific immune recruitment; in selected cold-tumor contexts, upstream lymphoneogenic induction through emerging pathways such as IL-33–ILC2 may become one candidate strategy, but it will require spatial confirmation of TLS maturity and antitumor function. A spatial framework does not replace classical pathology; it completes it by turning TLSs from descriptive histological objects into measurable immune microenvironmental units. The immediate next step is prospective validation of a minimal TLS panel across defined treatment contexts, with pre-specified scoring rules, harmonized pathology criteria, and longitudinal sampling before and during therapy.
